# Which Depressive Symptoms and Medication Side Effects Are Perceived by Patients as Interfering Most with Occupational Functioning?

**DOI:** 10.1155/2012/630206

**Published:** 2012-05-02

**Authors:** Raymond W. Lam, Erin E. Michalak, David J. Bond, Edwin M. Tam, Auby Axler, Lakshmi N. Yatham

**Affiliations:** Mood and Anxiety Disorders Program, Department of Psychiatry, Mood Disorders Centre, UBC Hospital, University of British Columbia, Vancouver, BC, Canada V6T 2A1

## Abstract

*Background*. Major depressive disorder (MDD) is associated with significant impairment in occupational functioning. This study sought to determine which depressive symptoms and medication side effects were perceived by patients with MDD to have the greatest interference on work functioning. *Methods*. 164 consecutive patients with MDD by DSM-IV criteria completed a standard assessment that included a self-rated questionnaire about the degree to which symptoms and side effects interfered with work functioning. *Results*. The symptoms perceived by patients as interfering most with work functioning were fatigue and low energy, insomnia, concentration and memory problems, anxiety, and irritability. The medication side effects rated as interfering most with work functioning were daytime sedation, insomnia, headache, and agitation/anxiety. There were no differences between men and women in symptoms or side effects that were perceived as interfering with work functioning. *Limitations*. This was a cross-sectional study; only subjective assessments of work functioning were obtained; the fact that patients were using varied medications acts as a potential confound. *Conclusions*. Specific depressive symptoms and medication side effects were perceived by patients as interfering more with occupational functioning than others. These factors should be considered in treatment selection (e.g., in the choice of antidepressant) in working patients with MDD.

## 1. Introduction

Unipolar major depressive disorder (MDD) is among the most common and disabling medical conditions. Many epidemiological studies have demonstrated the high prevalence of MDD in the general population. For example, the Canadian Community Health Survey (CCHS) recently reported a one-year prevalence rate of 4.5% for MDD, indicating that over 1.2 million Canadians suffer significant distress and impairment in functioning due to mood disorders [1]. Similar statistics are found for Europe [2] and the United States [3]. Depression is currently the fourth leading medical condition contributing to global burden of disease and is estimated to rise to second by the year 2030 [4].

Given the high prevalence of MDD, increasing attention is now being paid to the economic costs of depression. The economic burden is, in part, attributable to individuals with depression being unable to work, or absenteeism. For example, one study reported that workers with MDD missed an average of 32 days of work in a 12-month assessment period [5], while another found that about 30% of work disability claims in Canada were attributed to mental illness, predominant depression, and other mood disorders [6]. However, the greater proportion of the total economic burden of MDD lies in reduced productivity, or presenteeism, in which the depressed individual remains in the work setting but with productivity suffering both in quality and quantity [7, 8]. In Canada, the economic costs of depression-related presenteeism alone are estimated at over $5 billion annually [9].

The CCHS also found that, in 2002, 79% of people experiencing MDD in the previous year reported some interference with their work functioning [5]. It should not be surprising that depression is associated with such significant occupational impairment. The constellation of core symptoms of depression includes both physical (decreased energy, sleep disturbance) and cognitive (reduced interest and motivation, difficulty with concentration and attention) symptoms that would be expected to impair functioning in all types of work.

Several studies have explored which depressive symptoms predict occupational impairment. Some specific symptoms, such as low energy/fatigue, psychomotor disturbance, and low interest/pleasure [10], and difficulty concentrating/fidgety and feeling tired/sleep disturbance [11], have been found to predict impairment in work productivity. However, these studies examined nonclinical populations or combined clinical and nonclinical subjects. As well, while they statistically correlated self-rated symptom severity to work impairment, these studies did not solicit the opinions of subjects about which symptoms most affected their work performance, thus missing a potentially important aspect of study. In fact, there is little available information about the clinically depressed individual's subjective understanding of symptomatic interference with occupational functioning. The observed relationship between depressive symptoms and work impairment can also be confounded by treatment, which has not been examined in previous studies. Side effects (e.g., sedation, nausea, insomnia, etc.) associated with antidepressant medications may also adversely affect work functioning, even if mood and other depressive symptoms improve.

In this study, we sought to determine which depressive symptoms and medication side effects were subjectively experienced by individuals with MDD as most impairing their work functioning. Because of some studies showing differences between men and women in the experience of depressive symptoms [12], occupational stress [13, 14], and occupational impairment [15–17], we also examined the role of gender.

## 2. Methods

### 2.1. Subjects

Consecutive patients attending a Mood Disorders Clinic at a university teaching hospital completed several questionnaires as part of their initial assessment. Patients were referred from primary care physicians and from community psychiatrists. Clinical assessments were conducted by board-certified psychiatrists. Diagnoses were assigned according to DSM-IV criteria based on clinical interviews supplemented by a symptom check list and all available medical information. Inclusion criteria for this study included a DSM-IV diagnosis of major depressive disorder and a Quick Inventory of Depressive Symptomatology, Self-rated (QIDS-SR) [18] score of 5 or higher, indicating clinically significant depressive symptoms. Patients with bipolar disorder, or who did not do paid or volunteer work (outside the home), were excluded. This study was approved by the Clinical Research Ethics Board of the University of British Columbia.

### 2.2. Assessment Measures

At their initial assessment, all patients completed the QIDS-SR [18], a validated self-rated scale to assess severity and type of depressive symptoms, and the Sheehan Disability Scale [19]. In addition, patients completed a questionnaire specifically developed for this study that included two questions. The first question was, “IN THE PAST WEEK, how have the following symptoms interfered with your ability to work? By work, we mean paid work if you are employed, schoolwork if you are a student, and housework if you are a homemaker.” Fifteen common symptoms of depression (including all symptom criteria for MDD) were listed, each rated on a 5-point Likert scale that included the following responses: “Did not have symptom,” “Not at all,” “Somewhat,” “Very much,” and "So much that I had to stop working."

The second question was, “Sometimes people have side effects to medications. IN THE PAST WEEK, how have the following side effects interfered with you [*sic*] ability to work? By work, we mean paid work if you are employed, schoolwork if you are a student, and housework if you are a homemaker.” Thirty six common side effects (based on a side effect rating scale used in clinical trials) were listed, each rated on a 5-point Likert scale that included the following responses: “Did not have side effect,” “Not at all,” “Somewhat,” “Very much,” and “So much that I had to stop working.”

The mean scores for these symptom and side effect items were examined by assigning values of 0, 1, 2, and 3, respectively, to the latter four Likert responses. In addition, for each item, we determined whether the respondent rated it as “clinically important” interference with work functioning, defined as a response of “Very much” or “So much that I had to stop working.”

### 2.3. Statistical Analysis

All results are reported as mean  ± standard deviations (SD). Parametric comparisons were conducted with *t*-tests, nonparametric comparisons with Friedman's tests, and chi-square tests with Fisher's test as appropriate. Because this was an exploratory study, no corrections were utilized for multiple comparisons. All analyses were conducted with SPSS v.16 [20].

## 3. Results

In a 4-month period, a total of 178 eligible subjects were screened at the clinic. Fourteen of the eligible patients did not complete one or more of the questionnaires, leaving a total of 164 patients with complete data.  Table 1 shows demographic and assessment information for the sample. There were no significant differences between men and women in any of the variables, including mean QIDS-SR and SDS scores. There were also no differences in mean scores on the 3 individual SDS items (data not shown).

Table 2 shows the summary of responses from the questionnaire about depressive symptoms and their effect on work functioning. Most of the symptoms were commonly experienced by patients, with the prevalence rate ranging from 98% of the sample endorsing low mood to 66% endorsing suicidal ideation. All symptoms were associated with some subjective interference with work functioning. However, the proportion of patients reporting clinically important interference in work functioning with individual symptoms varied, ranging from 59% (lack of motivation) to 19% (suicidal ideation). Clinically important impairment in work functioning was endorsed by more than half of the sample as associated with lack of motivation, low energy, low mood, feeling physically slowed down, and anxious/tense/nervous.

Table 3 shows the summary of responses from the question about medication side effects and interference with work functioning, in those side effects that were experienced by at least 10% of the sample. The most troubling side effects associated with clinically important interference with work functioning were daytime sleepiness, trouble sleeping, headache, and anxiety/agitation.

A series of analyses compared responses between men and women. For individual depressive symptoms, there were no significant differences between men and women in the mean scores of interference with work functioning. In the rates of clinically important interference with work functioning (either “Very much” or “So much that I had to stop working” responses for each item), only “Trouble with memory” was significantly different, with women reporting greater interference with functioning than men (45% versus 27%, resp., *χ* ^2^ = 4.6, df = 1, *P* < 0.04). A nonsignificant trend was observed for women to endorse “Feeling physically slowed down” as more interfering with functioning than men (57% versus 43%, resp., *χ* ^2^ = 2.9, df = 1, *P* < 0.09). There were no significant differences between women and men for any medication side effects, in either the mean scores of interference with work functioning or in rates of clinically important interference.

## 4. Discussion

This study shows that symptoms of depression are commonly perceived by patients as significantly interfering with their occupational functioning, but that different symptoms do so to different degrees. Inspection of our data at an item level identifies three major symptom clusters that interfere most with functioning at work: anergia (lack of motivation; low energy; feeling physically slowed down; sleepy during the day), tension (anxious/tense/nervous; irritability/anger), and cognitive difficulty (trouble concentrating; trouble with memory). Clinically significant work impairment was endorsed in at least one of the individual symptoms within these symptom clusters by 66%, 54%, and 52%, respectively, of patients in this study. Some depressive symptoms were less likely to interfere with work functioning. For example, the symptom of “suicidal thoughts” was experienced by 66% of patients, but was rated by only 19% as clinically interfering with occupational functioning.

These results are generally consistent with those from other studies that have examined the effect of individual depressive symptoms on work productivity. In contrast to our study, in which patients self-reported the degree of work impairment from individual symptoms, other studies correlated scores from patient-rated scales of symptom severity with scales assessing work productivity. For example, Sanderson and colleagues [10] studied a nonclinical sample of 431 employees at 10 Australian call centres and examined depressive symptoms endorsed on a rating scale (the Patient Health Questionnaire, PHQ-9) and effects on work productivity, as measured by the work limitations questionnaire (WLQ) [11, 21]. Three symptoms (tired or little energy, little interest or pleasure, and psychomotor disturbance) were found to be significant predictors for presenteeism, while symptoms of low mood, sleep disturbance, and appetite disturbance were not predictive of work impairment. Our results (in patients with MDD) are very similar to theirs (in a nonclinical sample), with the exception that low mood and sleep disturbance were both identified by patients as interfering with work functioning in the current study.

Similarly, in a sample of patients screened at a primary care clinic, Lerner and colleagues [11] examined the relationship of some composite symptoms of depression (measured by ratings from the PHQ-9) to productivity loss (measured by the WLQ). The sample of 389 employed people included 246 who were depressed: 64 with dysthymia, 89 with MDD, and 93 with double depression. That study examined two specific symptom clusters: concentration/fidget (comprised of 2 items on the PHQ-9: difficulty concentrating and psychomotor change (fidgety or moving too slowly)) and tired/sleep problems (comprised of 2 items on the PHQ-9: feeling tired and having difficulty sleeping). Both symptom clusters were associated with significant loss of productivity, and the cluster of tired/sleep problems was also associated with days missed from work.

Antidepressant medications are widely used to treat working people with MDD. For example, in a sample of employees on depression-related short-term disability, 58% were prescribed antidepressants [22]. Our study found that many medication side effects are endorsed by patients as interfering with work functioning. The most troublesome side effects were daytime sedation, insomnia, headache, and anxiety/agitation. We note that these side effects should be considered as nonspecific, since the patients were taking different antidepressants and some were on multiple medications. Some medication side effects were commonly experienced by patients but were not associated with impairment in work functioning, for example, sexual side effects were endorsed by 32% of the sample, but 0% found these to be associated with clinically significant interference at work.

Some studies have found differences between men and women in depressive symptomatology and effects on work functioning, while others have not. For example, women have been found to have more work absence days than men [15]. Our results showed little effect of gender on self-perceived work interference, whether from depressive symptoms or from medication side effects. Only “trouble with memory” was reported by more women than men as interfering with work functioning, and this may have been a type I error since there was no statistical correction for multiple comparisons.

This study has a number of limitations, including the use of a cross-sectional design, the self-report nature of the data without objective assessment of occupational performance, the varied nature of work experienced by respondents, and the use of multiple and varied medication regimens by the patients. We also did not have information on whether patients were engaged in psychosocial treatments (e.g., cognitive behavioural therapy) that may affect work functioning. Nonetheless, there is clear clinical relevance to our findings. First, since numerous depressive symptoms are perceived by patients to be associated with work impairment, occupational functioning should be routinely assessed (via standardized, validated assessment scales) in the management of people who are clinically depressed. Second, treatment for MDD in working patients should address the symptoms that interfere most with work functioning, including anergia, tension, and concentration difficulty. Monitoring of symptoms and functioning during treatment is also important, to ensure that work functioning returns to premorbid status and is not affected by residual symptoms of depression. Finally, since the side effect profiles for individual antidepressants are quite different [23], the choice of medication for working patients should take into account those side effects that most impair work functioning. Antidepressants that minimize these side effects (daytime sedation, insomnia, headache, and anxiety/agitation) should be the preferred options for working patients with MDD.

In summary, patients with MDD report that the depressive symptom clusters of anergia, tension, and concentration difficulty are most interfering with occupational functioning, with no differences found between men and women. In the clinical management of working patients with MDD, it will be important to ensure that these symptoms are addressed by treatment. In addition, if pharmacotherapy is used, medications should be chosen to avoid those side effects (sedation, insomnia, headache, and anxiety/agitation) that are reported by patients as most interfering with work functioning.

## Figures and Tables

**Table 1:** Demographic and assessment data on patients (*N* = 164) with major depressive disorder.

Variable (±SD)	Men (*N* = 57)	Women (*N* = 107)	Total (*N* = 164)
Age (years)	41.1 ± 12.0	41.7 ± 13.0	41.5 ± 12.6
Marital status (%) (married/single/ divorced/separated)	54/30/6/8	38/38/17/6	43/35/13/7
Number of episodes	3.0 ± 3.4	2.4 ± 5.4	2.7 ± 4.8
Duration of current episode (months)	6.8 ± 9.9	7.7 ± 7.1	7.3 ± 7.9
Number of psychotropic medications	1.9 ± 1.2	2.0 ± 1.1	2.0 ± 1.1
QIDS-SR mean score	14.5 ± 5.4	15.1 ± 5.3	14.9 ± 5.3
SDS mean score	7.3 ± 2.4	7.3 ± 2.3	7.3 ± 2.3
Number of work days missed in past month	10.4 ± 12.0	10.6 ± 12.7	10.5 ± 12.3

SD: standard deviation; QIDS-SR: quick inventory of depressive symptomatology, self-rated; SDS: Sheehan disability scale.

**Table 2:** Summary of responses (*N* = 164) from questionnaire relating symptoms to interference with work functioning.

Symptom	% of sample experiencing symptom	Mean score	SD	Clinically important interference^1^ (%)
Lack of motivation	93	1.78	0.91	59
Low energy	96	1.72	0.87	58
Low mood	98	1.68	0.88	55
Feeling physically slowed down	94	1.50	0.92	52
Anxious/tense/ nervous	96	1.55	0.90	50
Trouble concentrating	96	1.48	0.80	45
Sleepy during the day	88	1.31	0.85	40
Trouble with memory	93	1.31	0.77	39
Trouble sleeping at night	84	1.30	0.93	39
Feeling guilty/ashamed	88	1.24	0.97	38
Irritability/anger	91	1.26	0.91	36
Physical pain	76	1.18	1.10	35
Sleeping too much	80	1.26	1.00	31
Poor appetite	69	0.95	0.97	28
Suicidal thoughts	66	0.79	0.92	19

^1^ Defined as a response of “Very much” or “So much that I had to stop working” to the following question: “In the past week, how have the following symptoms interfered with your ability to work?”

SD: standard deviation.

**Table 3:** Summary of responses (*N* = 164) from questionnaire relating medication side effects (>9% prevalence) to interference in work functioning.

Medication side effect	% of sample experiencing side effect	Mean	SD	Clinically important interference^1^ (%)
Daytime sleepiness	19	1.87	0.88	74
Trouble sleeping	26	1.84	0.92	73
Headache	22	1.67	0.98	59
Anxiety/agitation	14	1.74	1.10	57
Nausea/stomach upset	17	1.18	1.02	29
Weight loss/gain	18	0.87	0.97	17
Diarrhea/constipation	20	0.82	0.85	15
Sexual problems	32	0.08	0.30	0

^1^ Defined as a response of “Very much” or “So much that I had to stop working” to the following question: “In the past week, how have the following symptoms interfered with your ability to work?”

SD, standard deviation.
